# “Microbial and immune modulation by 2’-fucosyllactose supplementation during gestation: a strategy to prevent food allergies”

**DOI:** 10.1080/19490976.2025.2523813

**Published:** 2025-06-26

**Authors:** Carole Brosseau, Anaïs Rousseaux, Marine Le Romancer, Marie Hélène Ropers, Virginie Lollier, Marion De Carvalho, Sébastien Barbarot, Marie Bodinier

**Affiliations:** a INRAE, UR BIA, Nantes, France; b Department of Dermatology, CHU Nantes, Nantes, France; c INRAE, UMR PhAN, Nantes, France

**Keywords:** Gut microbiota, metabolites, feto-maternal tissues, gut barrier. Human milk Oligosaccharides, food allergy, Developmental Origins of Health and Disease (DoHAD), regulatory B cells

## Abstract

Food allergies are linked to dysfunction in intestinal microbiota, the immune system and the intestinal epithelial barrier, leading to immune tolerance failure at birth. We hypothesized that a diet enriched with a Human Milk Oligosaccharide (HMO), the 2’-fucosyllactose (2’-FL), during pregnancy would enhance the establishment of these systems and protect the child against food allergy. We previously demonstrated that in our mouse model, 2’-FL protected offspring from food allergy, abrogating allergic symptoms and reducing associated biomarkers. This study investigated microbial, immune, metabolic and gut physiology in mothers, fetuses and offspring to understand the mechanisms behind this protection. Gestational 2’-FL supplementation significantly modified maternal gut microbiota and induced regulatory B cells, which were also seen in fetuses. Additionally, pups from 2’-FL supplemented mothers exhibited distinct microbiota, a strengthened intestinal barrier, and more regulatory B cells compared to control pups. Our results demonstrate that 2’-FL supplementation during gestation induces lasting beneficial changes, protecting offspring from food allergies.

## Introduction

Allergies are non-communicable diseases linked to our environment and our diet. Food allergy (FA) affects up to 10% of infants in some countries^1,2^ and is not only increasing in prevalence but also in severity, as evidenced by a huge increase in food-related anaphylaxis incidents over the last decade.^3,4^ FA symptoms can manifest from the first months of life and are associated with dysfunction of three critical systems: the immune system,^5,6^ commensal microbiota^7,8^ and epithelial barriers^9,10^ leading to a failure in the establishment of immune tolerance.

Emerging evidence indicates that the increasing prevalence of FA is linked to changes in gut microbiota composition and function. For example, infants with atopic dermatitis and FA have a decreased abundance of beneficial bacteria such as *Bifidobacterium breve, Bifidobacterium adolescentis, Faecalibacterium prausnitzii*, and *Akkermansia muciniphila*.^7,11^ In children allergic to cow’s milk and egg, a microbial dysbiosis is observed, characterized by increased *Lachnospiraceae* and *Ruminococcaceae* and decreased *Bifidobacteriaceae*.^12^ Furthermore, allergic patients exhibit significant differences in gut microbiota diversity compared to age-matched controls, with non-allergic controls having higher levels of *Prevotella copri* and short-chain fatty acids (SCFAs), which are correlated with protective effects against FA.^13^ To better understand how commensal bacteria regulate FA, Feehley *et al*. demonstrated that germ-free mice colonized with feces from healthy infants were protected against anaphylactic responses to cow’s milk allergens, whereas mice colonized with feces from FA infants were not.^14^ This protection was associated with the presence of specific bacterial species, such as *Anaerostipes caccae*, highlighting the crucial role of intestinal bacteria in regulating allergic responses to dietary antigens. The application of probiotics and the combination of probiotics with other dietary interventions to correct intestinal microbiota imbalances and regulate food allergy has become a research hotspot. However, the mechanisms by which the intestinal microbiota regulates food allergy and the efficacy of probiotics are still in the preliminary exploration stage, and there are no clear and specific conclusions.^15,16^

Microbiota–host interactions play a key role in regulating the immune system, which is also dysregulated in FA patients. FA is characterized by a failure of oral tolerance mechanisms and a T helper (Th) 2-skewed immune response, marked by the secretion of pro-inflammatory cytokines and antigen-specific immunoglobulins (Ig) E and IgG1.^6,17^ FA is also associated with reduced levels of regulatory T (Treg) and B (Breg) cells which are crucial for maintaining immune tolerance.^18–20^ Increasing the levels of these regulatory cells through adoptive transfer in mouse model^21,22^ or oral immunotherapies has shown efficacy in suppressing FA symptoms.^23,24^ Therefore, increasing the rate of regulatory cell subsets is particularly relevant to treat or prevent FA.

Moreover, individuals with FA often exhibit increased intestinal permeability, facilitating the passage of allergens through the intestinal barrier.^25^ This is associated with reduced expression of tight-junctions^9^ and impaired mucus layer function, which plays a protective role in the digestive tract by expulsing of luminal contents potential pathogens.^26,27^

The ontogeny and maturation of the immune system and intestinal barrier are modulated by the microbiota and are established during the first 1000 days of life.^28^ During fetal life, the mother can transfer immune cells by microchimerism^29^ and metabolites produced by her microbiota to the fetus,^30,31^ contributing to the concept of “trained immunity.”^32^ This concept is closely related to the Developmental Origins of Health and Disease (DOHaD), which posits that early-life environmental factors can impact long-term health outcomes, including the risk of allergies.^33^ Maternal diet during pregnancy and/or lactation is crucial for the establishment of the offspring’s microbiome and immune system.^34^

Human milk oligosaccharides (HMOs), which are abundant in breast milk, have been shown to shape the infant gut microbiota by selectively stimulating the growth of Bifidobacteria, modulate immune function, and support gut barrier integrity, supporting the potential of HMOs to provide health benefits in adults. In human breast milk, HMOs represent the 3rd most abundant constituent after lactose and proteins.^35,36^ In mouse models of FA, postnatal supplementation with 2’-FL was shown to decrease allergic symptoms, reduce intestinal mast cell frequency, and increase Treg levels in mesenteric lymph nodes and Peyer’s patches.^37^ Another study in a β-lactoglobulin induced milk allergic mouse model, demonstrated that 2’-FL supplementation during the 4 weeks of sensitization decreased β-lactoglobulin specific IgE and increased anti-inflammatory cytokines IL-10, TGF-β, and IFNγ, reduction allergic symptoms.^38^ In these studies, HMOs were used to treat FA or to reduce allergic symptoms but not before the induction of the disease to prevent FA.

Human studies have correlated HMOs presence in breast milk with allergy development, with mixed results.^39^ Some studies suggest certain HMO profiles may increase allergy risk^40,41^ while others indicate protective effects.^42^ Notably, infants fed 2’-FL-rich breast milk had a lower risk of developing IgE-associated eczema, especially those born by cesarean section and at high risk of allergies.^43^ This discrepancy may be due to the fact that maternal HMO composition varies widely. Thus, the allergy-protective efficacy of HMOs depends on complex interactions between multiple factors, including maternal secretor status, infant risk status, geographical location, and allergy phenotype.^44^ The addition of HMOs to infant formulas represents the most significant innovation in child nutrition in recent years. Indeed, recent advances in HMO synthesis have enabled large-scale industrial production, leading to significant progress in the fortification of infant formulas with 2′-FL. Clinical studies have demonstrated that supplementing infant formula with 2′-FL is well tolerated^45,46^ and leads to a systemic reduction in inflammatory cytokines,^47^ and fewer respiratory infections compared to those receiving control formula.^48^ Due to these promising health benefits in infants, 2′-FL supplementation has also been explored in clinical trials involving adults, confirming its good tolerability. Dietary supplementation with HMOs has been shown to beneficially modulate the gut microbiota, particularly by promoting the growth of beneficial *bifidobacteria*.^49^ 2′-FL was also studied in relation to various health conditions, including irritable bowel syndrome,^50^ obesity^51^ and allergies.^43^

Therefore, we hypothesized that a gestational supplementation with 2’-FL would promote specific microbial and immune imprinting and strengthen the intestinal barrier in pups, thereby protecting against FA. We already published in a murine model of FA that gestational 2’-FL-intake protected the offspring from developing FA with an abrogation of allergic symptoms and reduced associated biomarkers.^52^ Also, it significantly altered the composition of the maternal gut microbiota that gradually reverted to that of the control group post-supplementation and led to a microbial imprint and a reinforcement of the gut barrier in the pups.

In this study, we aimed to decipher the full mechanism by which antenatal intake of 2’-FL prevents allergies, using a mouse model where dams were exposed to 2’-FL during gestation followed by FA induction in their offspring. We analyzed not only the composition but also the potential function of the microbiota in both the mothers and pups through shotgun sequencing to assess potential microbial imprint. Additionally, the immune system of the dams, fetuses and offspring were also deeply characterized by flow cytometry to explore feto-maternal immune exchanges and the possibility of a lasting immune imprint. Finally, gut physiology parameters in both the mother and offspring were measure to evaluate the impact of 2’-FL supplementation and gut barrier integrity.

## Materials and methods

### Strategy of HMO supplementation and food allergy-induced protocol

Six-week-old BALB/cJRj mice were purchased from Janvier labs (Le Genest-Saint-Isle, France) and housed at constant temperature (22° C) and humidity (40–60 %) in a ventilated cage system under a 12:12 h light/dark cycle in the animal facility of INRAE BIA in Nantes. The protocol was approved by the Ethics Committee on Animal Experimentation of the Pays de la Loire region (CEEA-PdL n°06, accreditation number: 24994 and 34,572). Mice were fed either a standard-control diet (CT group) or a diet supplemented with 25 g of 2’-FL/kg of diet (2’-FL group) (Glycom, Denmark) (Serlab, France). It was demonstrated in both mice^53^ and humans^49^ that a daily intake of 2.5% enriched diet with 2’-FL significantly modifies the gut microbiota, notably by increasing the abundance of beneficial bacteria and is well tolerated. Based on these findings, we supplemented the diet with 25 g of 2’-FL per kg of food, achieving a final enrichment of 2.5%. The supplementation with 2’-FL started 1 week after mice arrival and during acclimation (2 weeks), mating (1 week), and gestation (3 weeks) (Figure S1). The composition of the food was developed to provide all the nutrients necessary during mouse gestation. After delivery, mothers and pups were fed a standard diet. After weaning (at 21 days of life), the female offspring from each group of mothers (CT or 2’-FL) were randomized to abrogate the litter-mate effects. Wheat-FA was induced in 4-week-old female pups born from mothers that received either a control diet (CT FA) or 2’-FL diet (2’-FL FA) as previously described by Rousseaux et al.,^52^ and detailed Figure S1. Pups born from mothers that received either a control diet or 2’-FL diet, but were nor sensitized to wheat nor challenged, were used as control (CT and 2’-FL). The sample size was calculated using the BiostaTGV website. Based on our recent publication, we estimated that a minimum temperature difference of 0.7°C between allergic and non-allergic pups would be considered clinically significant, with a standard deviation of 0.5°C. A two-tailed power test was performed with a Type I error rate of 5% and a power of 90%. The estimated sample size was 11 animals per group. The protocol was run twice, with 6 animals per group so a total of 12 animals per groups were used.

### Time-mated pregnancies and feto-maternal tissues collection

Female BALB/cJRj mice were time-mated with male BALB/cJRj. Forty-eight hours before mating, straw from the male cage was placed in the female cages to induce mouse estrus to optimize the chances of fertilization. Male studs were housed individually with 1–2 females overnight. Pregnant mice were sacrificed at 18 days of gestation (GD18). Gestational tissues (decidua, placenta, and uterus), maternal tissues (spleen), and fetal tissues (intestine, bone marrow) were collected. Preparation of single-cell suspensions was performed as mentioned by Brosseau et al.^54^

### Metagenomic analysis

Stool samples were disrupted and homogenized in tubes containing ceramic beads using a TissueLyser III (Qiagen) at high-speed. DNA was extracted using QIAamp DNA micro kit (Qiagen). Fragmentation of the extracted total DNA was performed using the FS DNA Library Prep Set kit (MGI Tech, RPC) according to the manufacturer’s instructions. After a first purification on magnetic beads, the ligation of adapters to each sample was performed. The generated libraries were then purified on magnetic beads. Library size was verified by capillary electrophoresis on at least 10% of the samples. After quantification by fluorimetry, the libraries were normalized at 1pmol and pooled before denaturation at 95°C for 3 min. The pool was circularized into a single-strand product before being purified and sequenced using the DNBSEQ-G400 technology (MGI Tech, RPC). Contributed sequences were analyzed with a dedicated bioinformatics pipeline developed by BIOFORTIS. Software and reference databases used in this processing are: 1/DeconSeq v0.4.3^55^ with Human Reference GRCh38, 2/FaQCs v2.08,^56^ 3/Centrifuge v1.0.4,^57^ 4/RefSeq Complete Genomes Archaea, Bacteria, Fungi, Protozoa, Viral (03/2021), and 5/HUMAnN2 v0.11.1^58^ with ChocoPhlAn and UniRef 90. For statistical analysis, the MiRKAT family of tests was used to assess overall association between taxonomic compositional profiles and treatment group.^59–61^ Bray-Curtis beta diversity score was used to quantify dissimilarity between compositional profiles at several taxonomic ranks and functionality types. Shannon alpha-diversity indices was computed at the species level for taxonomic data and at EC4 and pathway levels for EC and MetaCyc functionality outputs, respectively. Group comparisons for these indices were performed using Wilcoxon rank sum test. Assessment of microbiota components showing differential abundance between treatment groups was evaluated using a set of univariate statistical models, which capture different aspects of the data. This is in line with the recommendation from recent benchmarking.^62,63^ Tests were conducted in software R and included the following models: Linear mixed models with Gaussian errors applied to log-transformed relative abundances (lm), fitted with package lme4, Zero-inflated beta regression models applied on non-transformed relative abundances (zib), from package ZIBR, Zero-inflated negative binomial model on non-transformed relative abundances (zinb), from package glmmTMB, Deseq2, using package DESeq2. Individual p-values were corrected for simultaneous testing using the Benjamini–Hochberg procedure to control false discovery rate.^64^ This adjustment was applied independently at each taxonomic rank and functionality output. Unless stated otherwise, only components with a q-value smaller than 0.05 are reported. When more than 20 components satisfied this threshold, only significant components with a mean abundance log-fold difference larger than 2.0 were displayed to improve readability.

The pipeline was launched with host genome decontamination based on the human reference genome. This step was implemented to prevent the potential inclusion of human sequences originating from the handler in the microbiota analysis. The pipeline did not include host genome decontamination based on the mouse reference genome. Any reads mapping to the mouse genome, if present, would have been assigned to the “unclassified” category at the Kingdom level during read classification. The trimming and quality control was performed by FaQCs v2.08 with the following parameters: trimming of bases with quality below 20 (−q 20), discarding of reads with average quality below 30 (–avg_q 30), discarding of reads with 2 or more continuous base “N” (−n 2), discarding of reads with length less than 100bp (–min_L 100) and the other parameters were left as default

### Quantification of short chain fatty acids in stool samples

Short chain fatty acid (SCFA) concentrations were determined in stool samples by gas chromatography-mass spectrometry as fully described previously.^65^

### NMR-based metabolic fingerprints of amniotic fluid and fetus intestine

NMR analysis was performed in amniotic fluid and in fetus intestine according to the method used in Brosseau et al.^54^

### Flow cytometry

A total of 1.10^6^ cells from the different organs were transferred to 96-well plates and stimulated for 5 h with RPMI, 5% FCS, 1% Penicillin and Streptomycin, phorbol-12-myristate-13-acrylate (50 ng/ml, Sigma Aldrich, USA) and ionomycin (1 µg/ml, Sidma Aldrich, USA). Brefeldin A and monensin (1 mg/ml, BD Biosciences, USA) were also added 1 h after stimulation. Cells were labeled with surface markers specific of T helper lymphocytes (Th1, Th2, Th17, and Th1–17), regulatory T cells, B lymphocytes (memory, naive, transitional B cells and plasma cells), regulatory B cells (B10, CD25^+^ Breg and CD9^+^ Breg), common myeloid precusors and dendritic cells (cDC1, CD2, double positive cDC, and pDC). These all sub-populations and associated panel used were entirely described in the Table S1. For the intestine, B lymphocytes and regulatory B cells frequencies were investigated in the Peyer’s patches, DC frequencies were investigated in the Lamina propria and T helper and regulatory T cells frequencies were investigated in the IEL. For the spleen, MLN, bone marrow, fetal and gestational tissues, all these immune populations were investigated. Common myeloids precursors were investigated in the spleen of dams, bone marrow, fetal and gestational tissues only. Cells were fixed and permeabilized with a Fixation/Permeabilization Solution Kit (BD Biosciences) to perform intracellular labeling. Cells were analyzed by flow cytometry on a BD FACSCantoTM II Cell Analyzer (BD Biosciences). Data were acquired with Diva 8.0 software and analyzed with FlowJo Software v10 (TreeStar, Williamson Way, USA).

### Histological, permeability, and protein analysis of the intestine

Jejunum and proximal colon sections were stored in 4% paraformaldehyde (Biovalley, France) before being embedded in paraffin and stained in a hematoxylin phloxine saffron solution. Histological observations of intestinal integrity were realized with ZenBlue ® software. For the jejunum section, the number of intact villi on 2000 µm, the length and width of the villi were measured and the number of goblet cells per villi were counted. For the proximal colon section, the histological scoring was established based on the following estimation: 1) the mucosal breakdown, 2) muscle breakdown, 3) cellular infiltration. The number of crypt with mucus was also counted. Para- and transcellular permeability of jejunum and colon sections were measured *ex vivo* with the USSING chamber method (Physiological Instrument, EM8-C USSING system VCC MC8, software : Acquire & Analyze) as described by Clarke *et al*.^66^ Fluorescence measurements were realized with fluorescein–5.6 sulfonic acid (400 Da; 1 mg/ml, Sigma-Aldrich, USA) and Peroxidase from horseradish (40 kDA; 10 mg/ml, Sigma, USA) for paracellular and transcellular permeability respectively.^67^

### Quantification and statistical analysis

Univariate statistical analyses were performed using GraphPad Prism software, version 8.1 (La Jolla, CA, USA). The ROUT method was used to identify outlier. The normality (Shapiro test) and the homoscedasticity (Fisher test) were tested. For two group comparison, values were compared using either the T-test or Mann – Whitney test. For four group comparison, we performed a one-way Anova followed by a Dunn test as posttest with Bonferonni as correction test. A two-sided *p* < 0.05 was considered statistically significant (* = *p* < 0.05, ** = *p* < 0.01, *** = *p* < 0.001, **** = *p* < 0.0001). Results are expressed as the mean ± mean error (SEM).

Multivariate analyses on cellular and molecular immune parameters were performed with R (4.3.2) using RStudio (2023.12.1).^68^ Each set of data (lymphocytes B and/or T) associated to organs (femur, intestine, spleen, MLN) by females or by pups in utero, at 3 weeks or 6 weeks was considered by block. First the presence of missing data was detected with the package VIM.^69^ If missing data per columns and rows were less than 10%, an imputation step was performed by the package missMDA,^70^ otherwise data were cured to remove individuals with too many missing data (>10%). The missing data came from either insufficiently concentrated samples in cells or outliers. After curing the data, the analysis step was continued by a discriminant analysis consisting in identifying whether samples are discriminated by supplementation, allergy, or both by using package mixOmics.^71^ To present data with the same colors as the rest of the article, figures have been redrawn with ggplot2.^72^

## Results

### The composition and function of the maternal gut microbiota are modified by 2’-FL intake

#### Composition of the maternal gut microbiota

**Figure 1.**
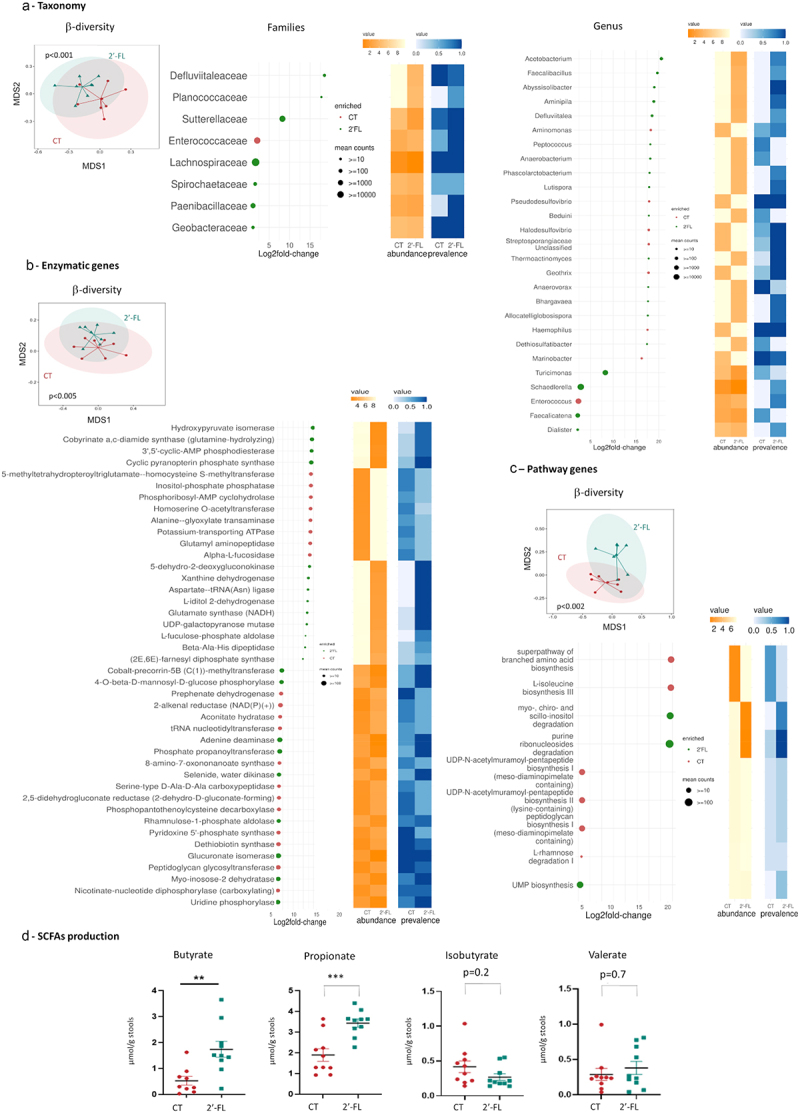
2’-FL supplementation during gestation modified the composition and the function of maternal intestinal microbiota. Shotgun analysis of stools of dams either fed a control diet (red) or a diet enriched in 2’-FL (green) at 18 days of gestation (a) at taxonomic level, (b) at the enzymatic gene expression level, (c) at the pathway gene expression level. (D) SCFAs concentration in the stools of dams either fed a control diet (red) or a diet enriched in 2’-FL enriched (green) at 18 days of gestation (**p < 0.005; ***p < 0.001).

To evaluate the impact of 2’-FL supplementation on the microbiota of pregnant mice, stools were collected at 18 days of gestation (GD18) (Figure S1) and analyzed by metagenomic sequencing. At GD18, the α-diversity was similar between the two groups (Figure S2) but the β-diversity differed significantly (Figure 1a). This was associated with a significant increase in abundance of the phyla Proteobacteria, Spirochaetes and Bacillota in the 2’-FL supplemented mother compared to CT mothers and a deceased abundance of the phyla Acidobacteria and Actinobacteria (Figure S2). Among Proteobacteria, the 2’-FL intake increased the *Sutterellaceae* and *Geobacteracea* families and among Bacillota, the *Defluvittaleaceae*, *Planococcaceae*, *Lachnospiraceae* and *Paenibacillaceae* families (Figure 1a). Finally, at the species level, 23 bacteria were increased in the 2’-FL group mainly from the *Clostridia* class and *Faecalibacillus* genus and 9 species were increased in the CT group (Figure 1(a) for genus and Figure S2 for species).

#### Function of the maternal gut microbiota

The metagenomic sequencing also allows the analysis of the potential functions of the microbiota by the analysis of Gene Ontology (GO) enrichment, enzymatic function (EC) and molecular pathway. GO enrichment analysis showed no differences between CT and 2’-FL mothers at GD18 (data not shown). Regarding the enzymatic function, the α-diversity was not different between CT and 2’-FL dams (data not shown), but the β-diversity was significantly different (*p* < 0.05) (Figure1(b)). At the EC4 level out of the enzymes that were more abundant, 13 were predominantly more abundant in the 2’-FL group compared to CT such as hydroxypyruvate isomerase, cobyrinate a,c-diamide synthase, 3’,5’-cyclic-AMP phosphodiesterase, cyclic pyranopterin phosphatase synthase. Eight enzymes were mainly more abundant in the CT groups compared to 2’-FL.

The pathway-based functional analysis of the metagenome highlighted no significant changes in α-diversity between CT and 2’-FL mothers at GD18 (data not shown) but a significant difference in β-diversity at GD18 (*p* = 0.001) (Figure 1(c)). was related to an increased purine ribonucleoside degradation, myo- chiro- scillo-inositol degradation and UMP biosynthesis in the 2’-FL groups compared to CT and an increased super pathway of branched amino acid biosynthesis and L-isoleucine biosynthesis in the CT groups compared to 2’-FL. Four other pathways were increased to a lesser extent in the CT group.

At GD18, the modification of microbiota composition was associated with an increased concentration of butyrate and propionate in 2’-FL stools compared to CT (butyrate: 1.74 ± 0.53 µmol/g vs 0.53 ± 0.38 µmol/g respectively, *p* = 0.003; Propionate: 3.43 ± 0.46 µmol/g vs 1.89 ± 0.76 µmol/g respectively, *p* = 0.0004) (Figure 1(d)). However, isobutyrate and valerate concentration were not different between both groups (*p* > 0.05).

### Gestational intake of 2’-FL leads to a microbial imprint in the pups

#### Composition of the pups gut microbiota at 3 weeks of age

**Figure 2.**
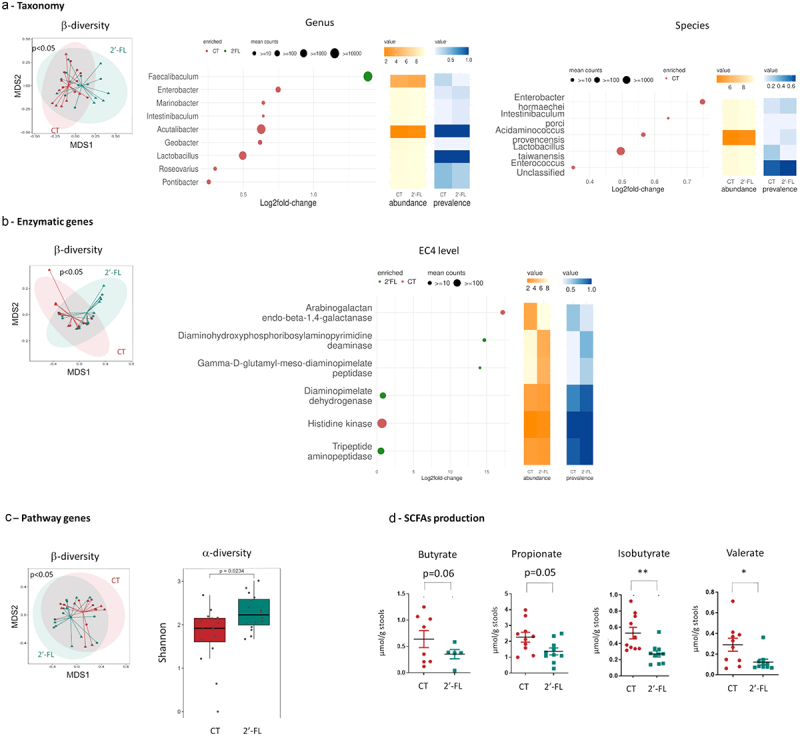
2’-FL supplementation during gestation modified the composition and the function of pups intestinal microbiota at 3 weeks of age. Shotgun analysis of stools of pups at 3 weeks of age issued from 2’-FL supplemented mothers (green) compared to CT mothers (red) (a) at the taxonomic level, (b) at the enzymatic gene expression level, (c) at the pathway gene expression level. (d) SCFAs concentration in the stools of pups at 3 weeks of age issued from 2’-FL supplemented mothers (green) compared to CT mothers (red) (*p< 0.05; **p< 0.005).

Similarly, to the mothers, the effect of 2’-FL supplementation during gestation on the modulation of the microbiota of the pups at 3 weeks of age was investigated by metagenomic sequencing. The α-diversity was similar between CT and 2’-FL groups (data not shown) but the β-diversity was different (*p* < 0.05) (Figure 2(a)) and was due to an overrepresentation of the *Faecalibaculum* genus in the 2’-FL group compared to CT and an underepresentation of the *Acutalibacter* and *Lactobacillus* genus and the *lactobacillus taiwanensis* specie.

#### Function of the pups gut microbiota at 3 weeks of age

We also investigated the effect of 2’-FL on the potential functionality of the microbiota by metagenomic. GO enrichment analysis showed no differences between CT and 2’-FL pups at 3 weeks of age (data not shown). At the EC level, the α-diversity between CT and 2’-FL pups was similar (data not shown) but the β-diversity differed significantly (*p* = 0.007) (Figure 2(b)). At the EC4 level, the diaminohydroxyphosphoribosylaminopyrimidine deaminase and the Gamma-D-glutamyl-meso-diaminopimelate peptidase were increased in the 2’-FL group but the Arabinogalactan endo-beta-1,4-galactanase was reduced. Finally, the pathway-based functional analysis of the metagenome highlighted significant changes in α-diversity and β-diversity between CT and 2’-FL pups (Figure 2(c)) but the differential analysis highlighted no specific pathway.

The functionality of the microbiota was also investigated by determining the concentration of SCFA in the stools. At 3 weeks of age, the concentration of isobutyrate and valerate were decreased in the 2’-FL group compared to the CT (isobutyrate: 0.27 ± 0.08 µmol/g vs 0.53 ± 0.19 µmol/g, *p* = 0.005; valerate: 0.12 ± 0.05 µmol/g vs 0.29 ± 0.15 µmol/g, *p* = 0.02 respectively) (Figure 2(d)). However, butyrate and propionate concentration were not different between both groups (*p* > 0.05).

### Gestational 2’-FL intake modified the composition and function of the gut microbiota in allergic pups at 6 week of age

#### Composition of the gut microbiota in allergic pups

**Figure 3.**
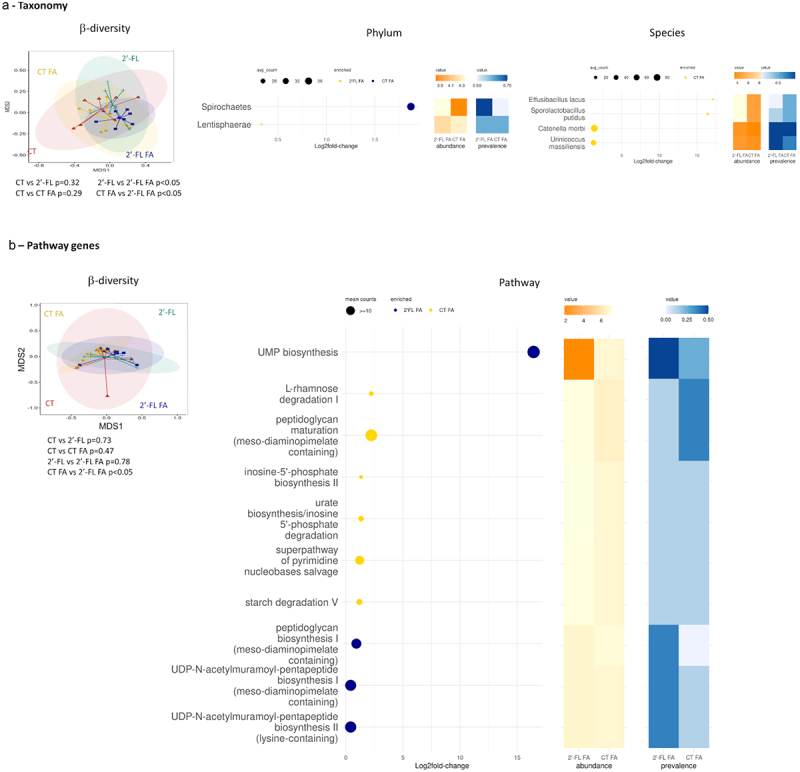
2’-FL supplementation during gestation modified the composition and the function of pups intestinal microbiota at 6 weeks of age. Shotgun analysis of stools at 6 weeks of age of non- and allergic pups from 2’-FL supplemented mothers (2’FL in green and 2’-FL FA in blue respectively) compared to non- and allergic pups from control mothers (CT in red and CT FA in yellow respectively) (a) at the taxonomic level, (b) at the pathway gene expression level.

Then, we determined if the difference in microbial composition in the pups at 3 weeks of age between 2’-FL and CT groups persisted until 6 weeks of age and after food allergy induction. Stools were collected at 6 weeks of age and analyzed by metagenomic sequencing. The α-and β-diversity was similar between CT and 2’-FL groups (not shown and Figure 3(a), respectively) but the relative abundance of some families such as *Lewinellaceae*, genus such as *Lewinella* and species such as *Prevotella ruminicola* were increased in the CT group (Figure S2B). Interestingly, the α-diversity was similar between CT FA and 2’-FL FA groups (data not shown) but the β-diversity was significantly different (*p* < 0.05) (Figure 3(a)). This difference was due to an increased abundance of the Spirochaetes phylum and increased abundance of *Sporolactobacillaceae putidus* and *Effusibacillus lacus* species in the CT FA group compared to the 2’-FL FA group. Moreover, the abundance of the Lentisphearae phylum was weakly increased in the 2’-FL FA groups compared to CT FA.

#### Function of the gut microbiota in allergic pups

Then, we analyzed the function of the microbiota by metagenomic sequencing in the pups at 6 weeks of age. GO and EC enrichment analysis showed no differences between CT and 2’-FL pups regarding the α- and β-diversity, nor between CT FA and 2’-FL FA (data not shown for GO and Figure S3a for EC). However, in healthy pups at the EC4 level, we observed an increase of 2 major enzymes: phosphonoacetaldehyde hydrolase and 2-phosphosulfolactate phosphatase in the 2’-FL group (Figure S3b). In allergic pups, at EC4 level an increase of 2 major enzymes were detected in the 2’-Fl group: 3,4-dihydroxy-2-butanone-4-phosphate synthetase and hydroxymethylglutaryl-CoA reductase (Figure S3b). The pathway-based functional analysis of metagenome highlighted no significant changes in α-diversity between CT, 2’-FL, CT FA and 2’-FL FA pups at 6 weeks of age (figure S3A) but a significant difference in β-diversity was observed between CT FA and 2’-FL FA groups (*p* < 0.05) (Figure 3(b)). This was mainly related to a huge increase of UMP biosynthesis in the 2’-FL FA group. Three other pathways were moderately increased in the 2’-FL FA group.

Moreover, SCFA concentrations in the stools were similar between the 4 groups (data not shown).

In conclusion, we demonstrated that 2’-FL intake during gestation reshape the maternal fecal microbiota composition and potential function allowing the implantation in the offspring of a microbiota with specific composition and function.

### Gestational intake of 2’-FL influences the concentration of metabolites in the fetus and its environment

Then, we investigated the effect of 2’-FL supplementation on the concentrations of metabolites in the amniotic fluid and in the fetal intestine. Samples were collected at GD18 and analyzed by nuclear magnetic resonance (NMR). Partial least squares discriminant analysis (PLS-DA) showed a clear separation between the control and 2’-FL-supplemented dams for both the metabolites in the fetal intestine and in the amniotic fluid (Figure S4a,b respectively). A total of 49 features were selected for the amniotic fluid and 124 for the fetal intestine, on the basis of the variable importance in the projection index (VIP >1) together with a Wilcoxon nonparametric test.

**Figure 4.**
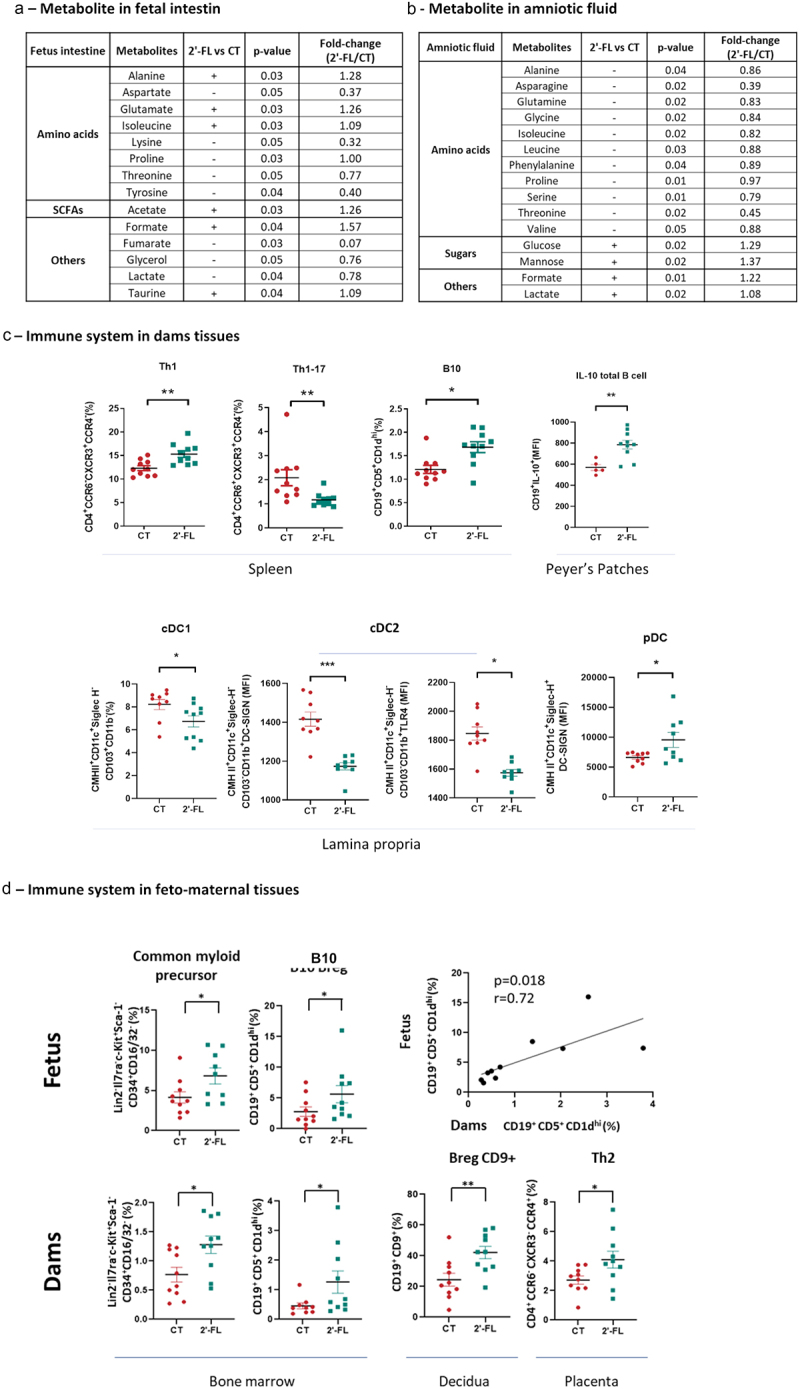
Gestational intake modulates the concentration of metabolites in the fetus and promotes tolerance in feto-maternal tissues. Table of discriminant metabolites in (a) the fetal intestine and (b) the amniotic fluid between dams fed a control or 2’-FL enriched diet. (c) frequency of Th1, Th1-like 17, B10 and cDC1 cells and the expression level (mean of fluorescence (MFI)) of IL-10 in B cells, DC-SIGN and TLR4 in cDC2, and DC-SIGN in pDC, in tissues (spleen, Peyer’s patches, lamina propria) of dams fed a control diet (red) or a diet enriched in 2’-FL (green). (d) Frequency of common myeloid precursor, B10 Breg, CD9^+^ Breg and Th2 cells in tissues (bone marrow, decidua and placenta) from dams and fetuses fed a control diet (red) or diet enriched in 2’-FL (green). Correlation curve of B10 frequency between dams and fetuses (*p< 0.05; **p< 0.005; ***pp < 0.001).

In the fetal intestine, the level of acetate and formate were significantly higher in the 2’-FL group compared to CT (acetate: Fold change 2’- FL/CT = 1.26, *p* = 0.03; formate: Fold change 2’-FL/CT = 1.57, *p* = 0.04) (Figure 4(a)). Moreover, it was observed that the concentrations of four other metabolites, belonging to the amino acid and citric acid cycle, were statistically higher in the intestines of fetuses from 2’-FL-supplemented dams compared to those from control dams. Conversely, 8 others metabolites were less concentrated in the intestine of fetus issued from 2’-FL-supplemented dams belonging also to the amino acids and citric acid cycle.

In the amniotic fluid of 2’-FL dams, 3 metabolites from the citric acid cycle (formate: fold change 2’-FL/CT = 1.22, *p* = 0.01; glucose: fold change 2’-FL/CT = 1.29, *p* = 0.02; Lactate: fold change 2’-FL/CT = 1.08, *p* = 0.02) were found in higher concentration compared to CT dams such as the monosaccharides mannose (fold change 2’-FL/CT = 1.37, *p* = 0.02) (Figure 4(b)). Conversely, 11 amino acids were less concentrated in the amniotic fluid of 2’-FL-supplemented mothers compared to CT.

### Gestational intake of 2’-FL promotes tolerance in maternal tissues

Next, we investigated the effects of 2’-FL supplementation on the immune system in the mother looking at the frequency of dendritic cells (gating strategy described in Brosseau *et al*.,^54^), B cells and T cells in systemic (spleen) and in the intestine (MLN, Peyer’s patches, Lamina propria, IEL) at GD18. In the spleen of dams fed 2’-FL, Th1 and Breg CD5^+^CD1d^hi^ (B10) were increased compared to CT (Th1: 15.3 ± 1.8% vs 12.3 ± 1.4%, *p* = 0.03; B10: 1.7 ± 0.3% vs 1.2 ± 0.2%, *p* = 0.01; for 2’-FL and CT respectively) (Figure 4(c)). Conversely, the frequency of Th1–17 was reduced in 2’-FL compared to control (1.2 ± 0.2% vs 2.1 ± 0.7% respectively, *p* = 0.01). The frequency of all the conventional B cell subtypes, Th2, Th17 and Treg were similar between both groups (data not shown).

The effect of 2’-FL supplementation was also determined locally in the intestine. In the Peyer’s patches, in the 2’-FL group, the Mean Fluorescence Intensity (MFI) of IL-10 in total B cell was higher compared to CT (784 ± 95 AU vs 570 ± 51 respectively, *p* = 0.004) (Figure 4(c)). On the contrary, 2’-FL intake had no effect on the frequency of any T cell subtype in the IEL (data not shown). In the MLN, the 2’-FL supplementation had no effects on B and T cell sub-types frequency (data not shown). The effects of 2’-FL supplementation on DC frequency and the expression of DC-SIGN and TLR4 on their surface was investigated in the Lamina propria. The rate of cDC1 was decreased in the 2’-FL group compared to CT (6.7 ± 1.4% vs 8.2 ± 0.1%, respectively, *p* = 0.01) (Figure 4(c)). The MFI of DC-SIGN was decreased on cDC2 cells in the 2’-FL group compared to the CT (1173 ± 35 vs 1416 ± 81 respectively, *p* = 0.0002) associated with a decreased MFI of TLR4 (1574 ± 48 vs 1846 ± 101 respectively, *p* = 0.0001). Conversely, the MFI of DC-SIGN was increased on pDC cell in the 2’-FL group compared to CT (9537 ± 3050 vs 6614 ± 754 respectively, *p* = 0.03).

### Gestational intake of 2’-FL promotes tolerance in feto-maternal tissues

Then, the effect of 2’-FL supplementation during gestation on the immune system of the gestational tissues of the mother and the fetus at GD18 was determined. First, we analyzed the effect of 2’-FL on hematopoïetic stem cells (HSC) and progenitor cell abundance in the fetal hind leg and dam femur as we previously described in Brosseau *et al*. for biomarkers and gating strategy.^54^ The frequencies of all HSCs and progenitor cells were similar between mothers supplemented or not with 2’-FL and between fetuses exposed or not to 2’-FL *in utero* (data not shown) except the frequency of CMPs (Lin2^−^Il7ra^−^c-Kit^+^Sca-1^−^CD34^+^CD16/32^−^) that was higher both in the femurs of dams and fetus exposed to 2’-FL (dams: 0.7 ± 0.12% vs 1.28 ± 0.14% for CT and 2’-FL, *p* = 0.01; fetus 4.1 ± 0.7% vs 6.8 ± 1% for CT and 2’-FL, *p* = 0.03) (Figure 4d).

The effects of 2’-FL supplementation on the frequency of total B cells, plasma cells, memory, naive and transitional B cells, B10, and Breg expressing CD9 or CD25 were assessed in the bone marrow, uterus, decidua, and placenta from mothers and from the cord blood, blood, intestine, and bone marrow of fetuses according to the gating strategy showed in Figure S5a. Only total B cells were detectable in the fetal tissues (cord blood, fetal intestine, blood) and were similar between the two groups (data not shown). In the femurs, the rate of B10 (expressing CD5^+^CD1d^hi^) was higher in the bone marrow of fetus exposed to 2’-FL than CT (2.78 ± 0.8% vs 5.62 ± 1.38% for CT and 2’-FL, *p* = 0.04) (Figure 4d). Similarly, the rate of B10 was also higher in the femurs of 2’-FL supplemented dams compared to CT (0.45 ± 0.1% vs 1.26 ± 0.37% for CT and 2’-FL, *p* = 0.03). Very interestingly, the rate of B10 in the femurs of the mothers was correlated with the rate of B10 in the fetal hind leg (*R* = 0.72, *p* = 0.01). No differences were detected for any B cell sub-types in any tissues except an increased level of Breg CD9^+^ in the decidua of supplemented dams (24.4 ± 3.8% vs 42 ± 4% for CT and 2’-FL, *p* = 0.007).

In these same tissues, the rate of T cell sub-types were investigated: total T cells, Th1, Th2, Th17, Th1-like Th17, and Treg cells (Figure S5b). No differences were detected for any T cell sub-types in any tissues (data not shown) except an increased level of Th2 in the placenta of 2’-FL supplemented dams (2.7 ± 0.29% vs 4.1 ± 0.57% for CT and 2’-FL, *p* = 0.004) (Figure 4d).

### Later in life, the tolerogenic environment in utero promoted by gestational intake of 2’-FL is maintained in pups at systematic level associated to a downregulation of inflammatory cells

#### Immune system of the pups at 3 weeks of age

**Figure 5.**
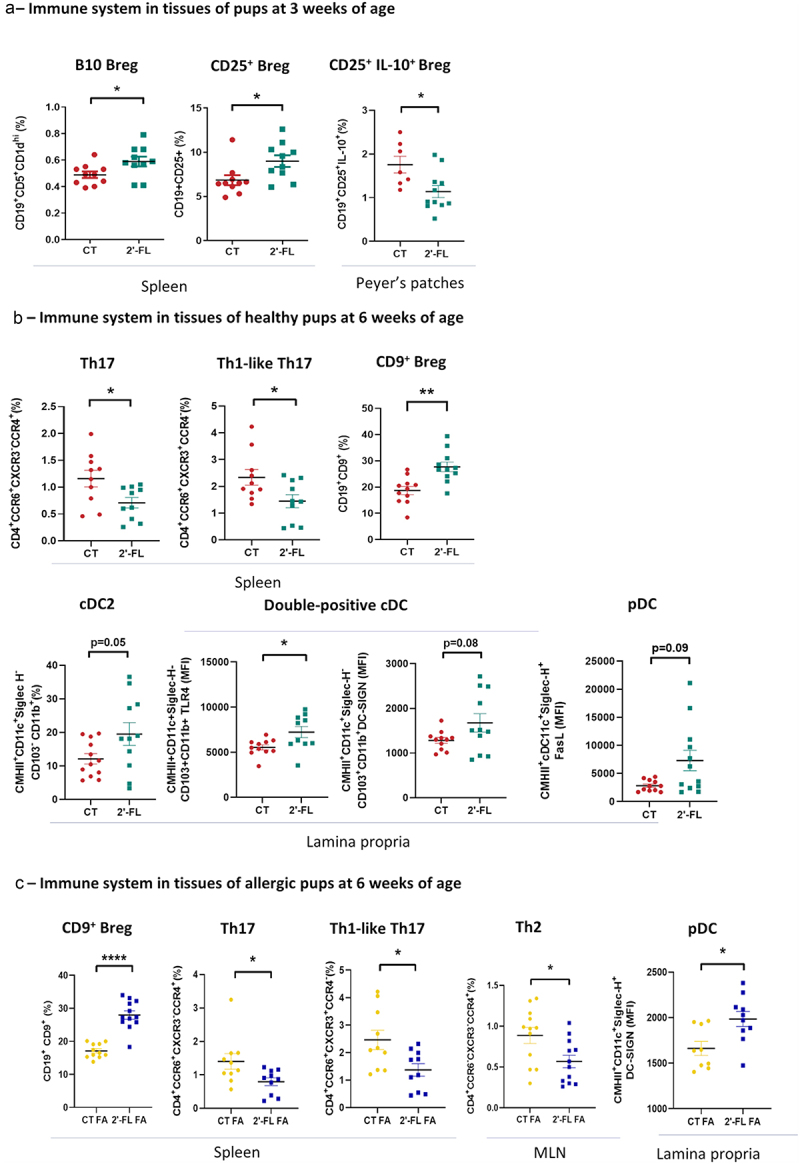
The tolerogenic environment established in utero by gestational 2’-FL intake is maintained in the pups whatever their allergic status. (a) Rate of B10, B CD25^+^ and B CD25^+^ secreting IL-10 in tissues (spleen and Peyer’s patches) of healthy pups from 2’-FL-supplemented mothers or control mothers at 3 weeks of age (b) Rate of Th17 cells, Th1-like 17 cells, CD9^+^, cDC2, double positive cDC expressing TLR4 or DC-SIGN, and pDC expressing FasL in tissues (spleen and lamina propria) of healthy pups at 6 weeks of age from 2’-FL-supplemented mothers (green) compared to those from control mothers (red). (c) Rate of Th2 cells, Th17 cells, Th1 like 17 cells, CD9^+^ Breg and pDC expressing DC-SIGN in tissues (spleen, lamina propria) of allergic pups at 6 weeks of age from 2’-FL-supplemented mothers (blue) compared to those from control mothers (yellow) (* *p*< 0.05; **p< 0.005, ****p< 0.0001).

Next, we investigated the effects of 2’-FL supplementation during gestation on the immune system of the pups at 3 weeks of age in the spleen for the systemic response and in the intestine for the local response. Interestingly, we observed a higher frequency of B10 and CD25^+^ Breg in the spleen of pups issued from supplemented mothers compared to non-supplemented one (B10: 0.59 ± 0.08% vs 0.49 ± 0.06%, *p* = 0.03; CD25^+^: 8.9 ± 1.7% vs 6.8 ± 1.1%, respectively, *p* = 0.04) (Figure 5a). However, in the Peyer’s patches, the frequency of CD25^+^ IL10^+^ Breg were decreased (1.1 ± 0.4% vs 1.8 ± 0.4%, *p* = 0.01). The frequency of the other B cell sub-types, T cells and DC cells were similar in the spleen, the MLN and the intestine between CT and 2’-FL offspring (data not shown).

#### Immune system of the pups at 6 weeks of age

Similarly to the pups at 3 weeks of age, the effects of gestational intake of 2’-FL on the immune system of the healthy pups at 6 weeks of age. In the spleen, we observed an increased rate of CD9^+^ Breg in 2’-FL groups compared to CT (27.7 ± 4.3% vs 18.7 ± 3.9, *p* = 0.0013 respectively) (Figure 5b). Conversely, the pro-inflammatory populations Th17 and Th1-like Th17 were decreased in 2’-FL groups compared to CT (Th17: 0.76 ± 0.27% vs 1.16 ± 0.38% respectively, *p* = 0.02; Th1-like Th17: 1.45 ± 0.62% vs 2.34 ± 0.69%, *p* = 0.03). In the Peyer’s patches, in the IEL and in the MLN, the frequency and the level of cytokine secretion of all sub-types of B and T lymphocytes investigated were similar among CT and 2’-FL groups (data not shown). Moreover, in the Lamina propria, the cDC2 frequency was increased in 2’-FL pups compared to CT (19.51 ± 8.75% vs 12.1 ± 3.95% respectively, *p* = 0.05). Also, the TLR4 and DC-SIGN cell surface expression on double positive cDC were increased in 2’-FL pups compared to CT (TLR4: 7236 ± 1558 MFI vs 5546 ± 563 MFI respectively, *p* = 0.01; DC-SIGN 1676 ± 418 MFI vs 1283 ± 147 MFI, respectively, *p* = 0.08) such as the expression of FasL on pDC (7284 ± 3619 MFI vs 2792 ± 843, respectively, *p* = 0.09) (Figure 5b).

Then, we characterized the effects of 2’-FL supplementation during gestation on the immune system of FA pups at 6 weeks of age. Similarly, to the 2’-FL pups, we observed an increased frequency of CD9^+^ Breg in the spleen of 2’-FL FA pups compared to CT FA (28 ± 3.3% vs 17.1 ± 1.8% respectively, *p* < 0.0001) (Figure 5c), and this was associated with a tendency to increase secretion of IL-10 (data not shown). Also similarly to the 2’-FL pups, the pro-inflammatory populations Th17 and Th1-like Th17 were decreased in 2’-FL FA groups compared to CT FA (Th17: 0.79 ± 0.3% vs 1.4 ± 0.48% respectively, *p* = 0.02; Th1-like Th17 1.37 ± 0.62% vs 2.46 ± 0.92%, *p* = 0.01). Very interestingly, the rate of Th2 was decreased in the spleen of 2’-FL FA pups compared to CT FA (0.57 ± 0.22% vs 0.89 ± 0.28%, respectively, *p* = 0.01). Finally, in the MLN, the expression of DC-SIGN on pDC was higher in 2’-FL FA compared to CT FA (1984 ± 175 MFI vs 1661 ± 172 MFI respectively, *p* = 0.01).

In conclusion, we demonstrated that 2’-FL supplementation during gestation promote the rate of Breg in the mother, the fetus, and the pups inducing the establishment of the immune tolerance.

### Gestational 2’-FL intake is associated with a gut physiological imprint in the pups

#### Maternal intestinal physiology

**Figure 6.**
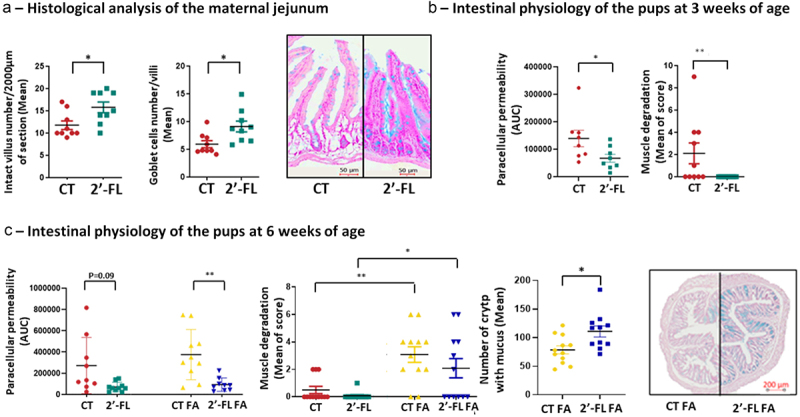
2’-FL supplementation during gestation reinforces the intestinal barrier in dams and offspring. (a) Histological analysis (villi number and goblet cells number) and histological section of jejunum from control dams (red) or dams fed a 2’-FL enriched diet (green). (b) Paracellular permeability of the jejunum and muscle degradation of the colon of pups at 3 weeks of age issued from 2’-FL supplemented mothers (green) or not supplemented (red). (c) Paracellular permeability of the jejunum, colonic muscle integrity, number of crypt with mucus in the colon and colonic histological section of non- and allergic offspring issued from 2’-FL supplemented mothers (green and blue respectively) compared to non- and allergic offspring issued from control mothers (red and yellow respectively) at 6 weeks of age (* *p*< 0.05; **p< 0.005, ***p< 0.001).

The effects of 2’-FL supplementation on paracellular intestinal permeability were determined with the USSING chambers methods on the jejunum and the colon of the mothers collected at GD18. No difference was observed between CT and 2’-FL groups both for the jejunum and the colon (data not shown). To assess the effects of 2’-FL on the intestinal epithelial barriers morphology, the jejunum and the proximal colon were collected from the mothers at weaning to perform a histological analysis. The histological analysis of the jejunum showed that 2’-FL intake induces an increase in the number of goblet cells and amount of mucus (in blue on the histological section) and the number of intact villi (in pink on the histological section) on 2000 μm of section compared to CT group (9.1 ± 2.1 vs 5.9 ± 1.4 goblet cells per villi, *p* = 0.01 and 15.7 ± 3 vs 11.7 ± 2.3 intact villi on 2000 µm of section, *p* = 0.02 in 2’FL vs CT, respectively) (Figure 6a). 2’-FL supplementation had no effect on the morphology of the proximal colon compared to the control group (data not shown).

#### Intestinal physiology of the pups at 3 weeks of age

At 3 weeks of age, the histological analysis showed that the morphology of the jejunum was similar between the pups issued from 2’-FL supplemented mothers compared to CT mothers (data not shown). Interestingly, the paracellular jejunal permeability was reduced in pups issued from 2’-FL-supplemented mothers compared to the non-supplemented group (67265 ± 33061 AUC vs 139,187 ± 57909 respectively; *p* = 0.004) (Figure 6b). The transcellular jejunal permeability was similar (data not shown). Concerning the colon, the trans- and para-cellular permeability were similar between CT and 2’-FL pups (data not shown), but the score of integrity of the colonic smooth muscle was significantly reduced in 2’-FL pups (0 ± 0 vs 2.1 ± 2.32, *p* = 0.03) (Figure 6b).

#### Intestinal physiology of the pups at 6 weeks of age

The induction of FA in pups at 6 weeks of age had no impact on the paracellular jejunal permeability (374260 ± 185616 AUC vs 271,585 ± 202454 AUC for CT FA vs CT; *p* = 0.6) (Figure 6c). However, in FA-pups, the paracellular intestinal permeability was significantly decreased in 2’FL pups compared to CT pups (91915 ± 44388 AUC for 2’-FL FA, *p* = 0.004). No difference in paracellular colic permeability was observed between the four groups (data not shown).The histological analysis showed no difference in the morphology of the jejunum among the different groups of pups (data not shown). However, FA induction in pups led to the degradation of the colonic smooth muscle (3.08 ± 1.58 vs 0.5 ± 0.75 muscle degradation scoring for CT FA vs CT, *p* = 0.002) and pups born to 2’-FL supplemented mothers were not protected from this degradation (2.08 ± 2.1 vs 0.08 ± 0.15 for 2’-FL vs 2’-FL FA, *p* = 0.02) (Figure 6c). Moreover, in pups issued from 2’-FL supplemented mothers, the number of crypt with mucus in the colon was higher in FA-pups compared to CT FA pups (111 ± 24 crypts with mucus in the colon of 2’-FL FA vs 78 ± 19 crypts with mucus in the colon of CT FA, *p* = 0.01) such as the amount of mucus as observed in blue on the histological section.

In conclusion, we demonstrated that 2’-FL supplementation during gestation strengthens the gut permeability and favors gut integrity.

### Identification of immune biomarkers at cellular and molecular levels contributing to FA prevention in the pups by gestation intake of 2’-FL

**Figure 7.**
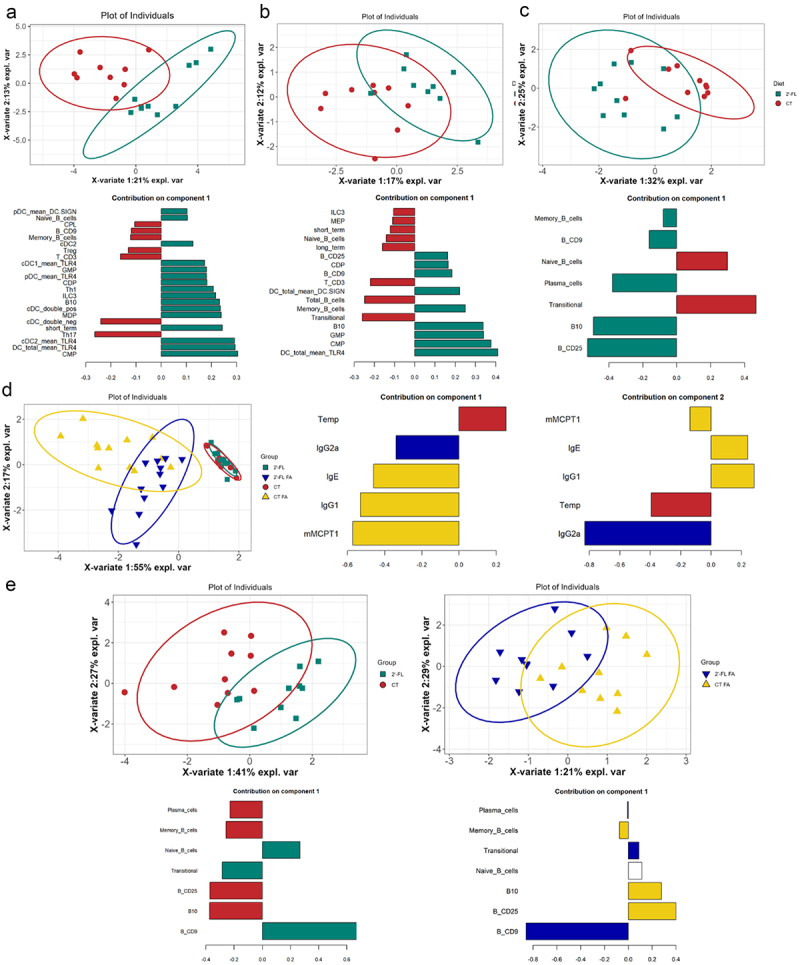
Multivariate statistical analysis of biomarkers contributing to FA prevention in the pups by gestation intake of 2’-FL. Sample plots and loading plot (component 1) of PLS-DA on immune biomarkers in a) dams bone marrow under control diet (red) or supplemented in 2’-FL (green), b) bone marrow of fetus from mother under control diet (red) or supplemented in 2’-FL (green), c) the spleen of pups at 3 weeks of age issued from mother under control diet (red) or supplemented in 2’-FL (green), d) the blood of allergic pups at 6 weeks of age issued from mother under control diet (yellow) or supplemented in 2’-FL (blue). e) the spleen of non-allergic pups issued from mother under control diet (red) or supplemented in 2’-FL (green) or allergic pups issued from mother under control diet (yellow) or supplemented in 2’-FL (blue), at 6 weeks of age. Contributions to components are colored according to the group in which the expression of the variable is maximal based on the median.

Finally, we performed a multivariate statistical analysis comparing all the cellular and molecular immune biomarkers (cells, cytokine secretion, and membrane expression of receptors) investigated in dams, fetuses and pups. In the bone marrow of the dams and the fetuses, PLS-DA showed a clear separation between the control and 2’-FL groups (Figure 7a,b). In dam bone marrow, this separation was mainly due to a higher rate of CMP and B10, and a higher expression of TLR4 on the surface of total DC and especially cDC2 in the 2’-FL group versus a higher rate of Th17 in the CT group (Figure 7a for component 1 and Figure S6a for component 2). Similarly, in the fetal bone marrow, the separation was mainly due to a higher rate of CMP, GMP and B10 and a higher expression of TLR4 on the surface of total DC in the 2’-FL group versus a higher rate of transitional cells in the CT group (Figure 7b for component 1 and Figure S6b for component 2). Interestingly, similar results were also found in the spleen of pups at 3 weeks of age. A clear separation between the CT and 2’-FL groups was due to a higher rate of B10 and B CD25 in the 2’-FL group and a higher rate of transitional cells in the CT group (Figure 7c and figure S6c). Regarding the clinical immune biomarkers of FA (Figure 7d), PLS-DA showed a clear separation between the FA groups associated with higher rate of IgG2A and no drop in rectal temperature after challenge in the 2’-FL-FA group versus a higher rate of mMCPT1, IgG1 and IgE in the CT-FA group. Finally, regarding the immune biomarkers in the spleen of both non-allergic and allergic pups at 6 weeks of age, CT and 2’-FL groups are well distinguished by stronger rate of B CD9 cells in the 2’-FL group in contrast to the CT groups (Figure 7e and Figure S6d,e for non-allergic and allergic pups, respectively).

## Discussion

Among the environment, the maternal diet may affect microbial, metabolic, and immune transfers from mother to child during gestation and lactation, which is a crucial window for proper establishment of the immune system, the microbiota, and the epithelial barriers.^73–75^ These three biological systems are known to be dysfunctional in allergy. We previously highlighted by a preclinical study that 2’-FL supplementation during gestation fully prevents the occurrence of FA in the offspring.^52^

Few preclinical studies have investigated the effects of antenatal nutritional strategies on allergy prevention. In a previous study, we demonstrated that supplementation with the prebiotics GOS/Inulin reduced symptoms of food allergy.^76^ Similarly, Hogenkamp et al. reported that GOS/FOS supplementation during gestation reduced respiratory allergy symptoms in offspring.^77^ Unfortunately, in a human clinical study, maternal prebiotic (GOS/FOS) supplementation during pregnancy and lactation was not an effective strategy to prevent nor reduce infant eczema development.^78^ Also in clinic, high doses of vitamins C and E showed no benefit in improving respiratory symptoms in infants.^79^ Vitamin D supplementation during pregnancy has also shown no effect on the primary prevention of allergic diseases.^80^ Finally, while one meta-analysis found no effect of omega-3 on infant eczema,^81^ another indicated that omega-3 fatty acids supplementation during pregnancy may reduce the incidence of wheeze/asthma of children.^82^ Here, we demonstrate for the first time a complete abrogation of allergy symptoms through antenatal supplementation, and that 2’-FL is a significantly more effective preventive strategy than traditional prebiotics.

Despite the relevance of our mouse model of food allergy to wheat has been well documented and shown to closely mimic the specific IgE response of patients to wheat gliadins,^83^ this work still requires validation in a clinical study. Our study is based on animal models, which cannot fully replicate human physiology. Mice do not naturally produce 2’-FL. The composition of the murine microbiota differs from that of humans, even if some major species are shared. The mouse model is maintained in a controlled environment with a standardized diet and finally, the animals are genetically identical. We could consider validating our strategy in a human clinical study. Although 2’-FL is currently used to fortify infant formula, is being investigated in several clinical trials in adults, promotes the growth of intestinal bacteria beneficial for health and is well tolerated.^45,46,49^ Given these findings, a clinical trial investigating the effects of 2′-FL supplementation during pregnancy to prevent allergy development in infant appears feasible. This could follow a similar approach to our ongoing PREGRALL study (ClinicalTrials.gov: NCT03183440), which is currently assessing the impact of antenatal supplementation with GOS/Inulin prebiotics on allergy development in at-risk infants.^84^

However, it is thanks to murine models that we can gain deep mechanistic insights, and we have demonstrated that this nutritional strategy fosters the setup of these biological systems in the pups by 1) reshaping the maternal fecal microbiota composition and function allowing the implantation in the offspring of a microbiota with specific composition and function 2) promoting the rate of Breg in the mother, the fetus, and the pups inducing the establishment of the immune tolerance, 3) strengthening the gut permeability and favoring the gut integrity.

Regarding the maternal microbiota composition, we observed an increase in Bacillota, particularly of *Lachnospiraceae*, in maternal stool samples at the end of gestation following 2’-FL intake. This aligns with previous studies that reported similar effect in mice with human microbiota^85^ and in children.^86^ Additionally, we observed a reduced abundance of the *Marinobacter* genus in 2’-FL supplemented mothers. This genus was also underrepresented in the gut microbiota of their pups at 3 weeks of age. At 3 weeks of age, we observed differences in microbiota composition and short-chain fatty acid profiles between the control and 2’-FL-treated groups, indicating that the treatment continued to leave an imprint on the microbiota. By 6 weeks, these differences in composition and SCFA levels were no longer detectable in healthy pups. This change coincides with the introduction of solid food, which is known to reshape the microbiota and may hide the effects of the initial treatment. However, at 6 weeks, in allergic individuals, a microbial imprint of 2’-FL treatment was still noticeable, as shown by differences in beta diversity, despite no observable changes in SCFA profiles. Under inflammatory conditions, the impact of 2’-FL on the microbiota remains detectable, particularly through an increase in UMP biosynthesis activity, suggesting a differential microbial response in the context of allergy. Other pathways were increased in the 2’-FL group such as the peptidoglycan biosynthesis I, and the UDP-N-acethylmuramoyl-pentapeptide biosynthesis I and II but to a lesser extent. Interestingly, the UMP synthesis was found increased both in 2’-FL mothers and 2’-FL FA pups. UMP serves as a source of uridine, which can be utilized in various biochemical processes – for instance, in the synthesis of glycoproteins – by acting as a donor of activated sugars through intermediates like UDP-glucose or UDP-galactose. It was studied for its effects on brain development and neuroplasticity, particularly in combination with other compounds such as docosahexaenoic acid and choline.^87^ It is sometimes used in dietary supplements to support memory, cognitive health, and recovery following neuronal injury. These findings highlight the strong impact of 2’-FL intake on both the functionality and specific relative abundance on the maternal microbiota, with potential protective effects extending to the next generation.

Still in the context of microbial modulation by 2’-FL, we demonstrated an increased concentration of lactate and formate in the amniotic fluid of 2’-FL supplemented mothers, as well as increased concentration of formate and acetate in the fetal intestine. This likely results from the fermentation of 2’-FL by the maternal gut microbiota, with metabolites migrating via the bloodstream into the amniotic fluid and then to the fetal intestine. Indeed, Zabel and colleagues demonstrated that fermentation of 2’-FL by the microbiota produces formate and lactate,^88^ and it has been shown that SCFAs can cross the placenta.^89^ Interestingly, we observed in the bone marrow of fetuses from 2’-FL supplemented mothers, an increased rate of B10. One hypothesis is that these Breg are induced by the increase of SCFA level observed in the amniotic fluid and the fetal intestine, as SCFA can differentiate conventional B cell into Breg.^76,90^ Another hypothesis is that these Breg originate from a microchimerism, where maternal Breg transfer to the fetus. This is supported by our observation of increased Breg rate in the bone marrow of 2’-FL supplemented dams which correlated with Breg rate in fetal bone marrow. Moreover, Stelzer *et al*., demonstrated in mice that maternal microchimeric cells are primarily located in the fetal bone marrow and play a functional role in promoting fetal immune development by inducing the preferential differentiation of HSCs into monocytes within the myeloid compartment.^29^

Our multivariate statistical immune analysis also revealed an increase in CMP in both maternal and fetal bone marrow along with a higher expression of TLR4 on the surface of DCs. This may result from a higher rate of CMP itself, a direct effect of 2’-FL bounding TLR on DCs,^53^ or higher rate of lipopolysaccharides secreted by the microbiota and induced by 2’-FL which also bounds to TLR on DCs.^91^ The higher expression of TLR4 on DCs might also be the origin of the higher rate of B10 observed in both maternal and fetal bone marrow.^92^ Finally, the same Breg were found in the spleen of the pups at 3 weeks of age showing the establishment of a persistent tolerogenic environment. These results highlight the fetal immune imprinting associated with tolerance induced by maternal 2’-FL supplementation which persist after birth.

The increase in the relative abundance of *Faecalibaculum* that we observed in the stools of pups at 3 weeks of age may contribute to the tolerogenic environment since this bacterial family was shown to have anti-inflammatory properties by inducing Treg.^93,94^ Therefore, given the effect of *Faecalibaculum* on the Treg response, we can assume that the Breg response can also be induced by this bacterial family. Our study also demonstrated a strengthening of the intestinal barrier in offspring at 3 weeks of age from 2’-FL supplemented mothers associated with a decrease in paracellular permeability. Moreover, *Faecalibaculum*, which is increased in pups microbiota, may play a role in the reinforcement of barriers since it is positively correlated with the expression of some tight junction proteins, contributing to the reduce paracellular permeability.^93,95^

This reinforcement may also have a fetal origin due to the increased concentration of lactate and formate observed in the amniotic fluid of 2’-FL supplemented dams. Both were shown to reinforced the intestinal barrier through the induction of tight junction protein expression.^96,97^ This suggests that the observed changes in the intestinal barrier in offspring may be also partly due to these metabolites present in the fetal environment. Thus, the strengthening of the intestinal barrier may be attributed to a direct effect of *Faecalibaculum*, or an indirect effect from metabolites. Because a small portion of HMOs are absorbed by intestinal cells,^98^ the strengthening of the intestinal barrier in the fetus may be also attributed to a direct effect of 2’-FL on gut epithelial cells by upregulation of tight junction proteins. Indeed HMOs, and particularly 2’-FL, were shown in vitro to induce the expression of ZO-1, JAM-A, and claudin-8^99,100^ We also found improved colonic integrity and an increased mucus layer in pups from 2’-FL-supplemented mothers. By promoting intestinal cell differentiation, HMOs may contribute to the maturation of the intestinal barrier^101,102^ and reinforce gut defense by stimulating mucin production.^103^

Our work highlights potential cellular and molecular biomarkers – both microbial and immune – associated with the preventive effect of 2’-FL on allergy development. Importantly, our study is only observational and does not explore the underlying mechanisms. At this stage, the exact mode of action by which 2’-FL exerts its preventive effects remains unclear. Further mechanistic studies using dedicated protocols are needed to deepen our understanding. Our results support the notion that the maternal microbiota plays a central role in the prevention of food allergy through 2’-FL. However, it remains unclear whether this effect is mediated by maternal microbiota-induced modulation of the fetal immune system during pregnancy – potentially through trained immunity – or by the transmission of maternal microbes to the offspring at birth, which could shape early-life immune development. It is also possible that both mechanisms are required for the protective effect.

In conclusion, a gestational supplementation with 2’-FL is an effective strategy to prevent FA. We hypothesize this protection is mediated by 1) the vertical transfer of a specific microbiota during delivery fostering a tolerogenic environment and strengthening the intestinal barrier in pups, 2) the production of specific SCFA that cross gestational tissues, inducing Breg differentiation and leading to fetal immune imprint and intestinal barrier strengthening *in utero*, and 3) the synthesis of specific SCFA that induce Breg differentiation in the maternal bone marrow, with potential maternal to fetal transfer by microchimerism, leading to fetal immune imprint. All these hypotheses will be further investigated in our lab over the next few years, to decipher the specific involvement of immune and microbial imprinting in protection against food allergies.

## Supplementary Material

Supplemental Material

## Data Availability

The authors confirm that the data supporting the findings of this study are available within the article and its supplementary materials.
