# Supplementary material for: “Microbial and immune modulation by 2’-fucosyllactose supplementation during gestation: a strategy to prevent food allergies”
Source: Gut Microbes. 2025 Jun 26;17(1):2523813. doi: 10.1080/19490976.2025.2523813 (PMC12203853; doi:10.1080/19490976.2025.2523813)

Supplementary Figure

**Figure S1: Experimental study design of dams exposure to 2’-FL during gestation and food allergy induction in the offspring**. Mothers were fed a 2.5% 2’-FL enriched diet (2’-FL) or a control standard diet (CT) during gestation only and then, fed a standard diet. At 4 weeks of age, wheat-FA was induced in pups born from mothers that received either a control diet (CT FA) or 2’-FL diet (2’-FL FA). Pups were sensitized with 2 intraperitoneal injections of 10 μg of allergen-deaminated gliadins and aluminum hydroxyde as adjuvant, separated by 10 days. One week after the second intraperitoneal injections, pups were orally challenged with 20 mg of deaminated-wheat allergen by intragastric probe. Pups born from mothers that received either a control diet or 2’-FL diet, but were nor sensitized to wheat nor challenged, were used as control (CT and 2’-FL). The indicated samples were collected at different times during these protocols to perform the analysis


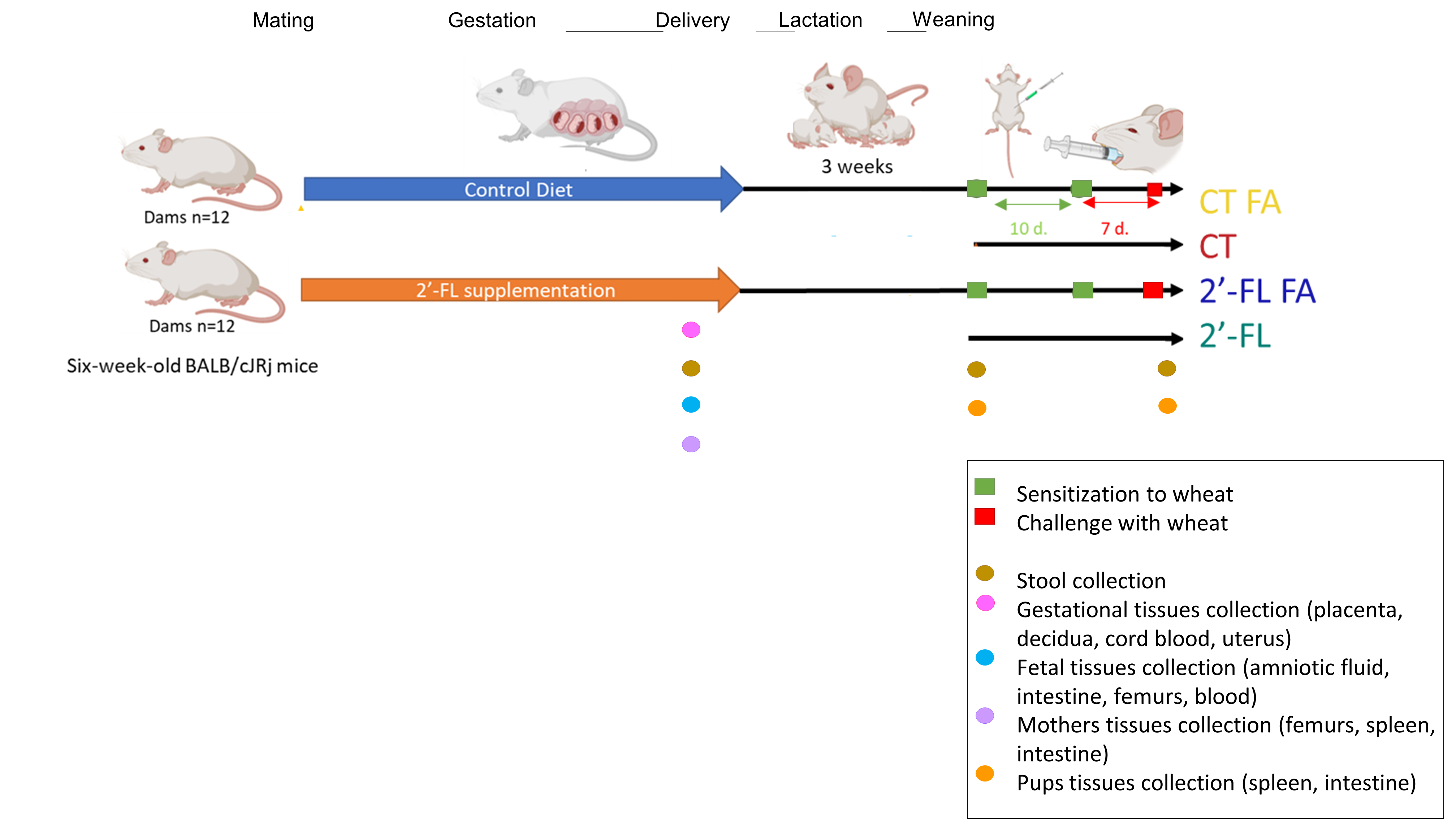


**Figure S2: Shotgun analysis of the maternal and pups gut microbiota. (A)** α-diversity, phylum and species composition of stools from dams fed a control diet (CT) or a 2’-FL enriched diet (2’-FL) at GD18. **(B)** Families, genus and species composition of stools from pups issued from dams fed a 2’-FL enriched diet (2’-FL) or fed a control diet (CT) at 6 weeks of age.


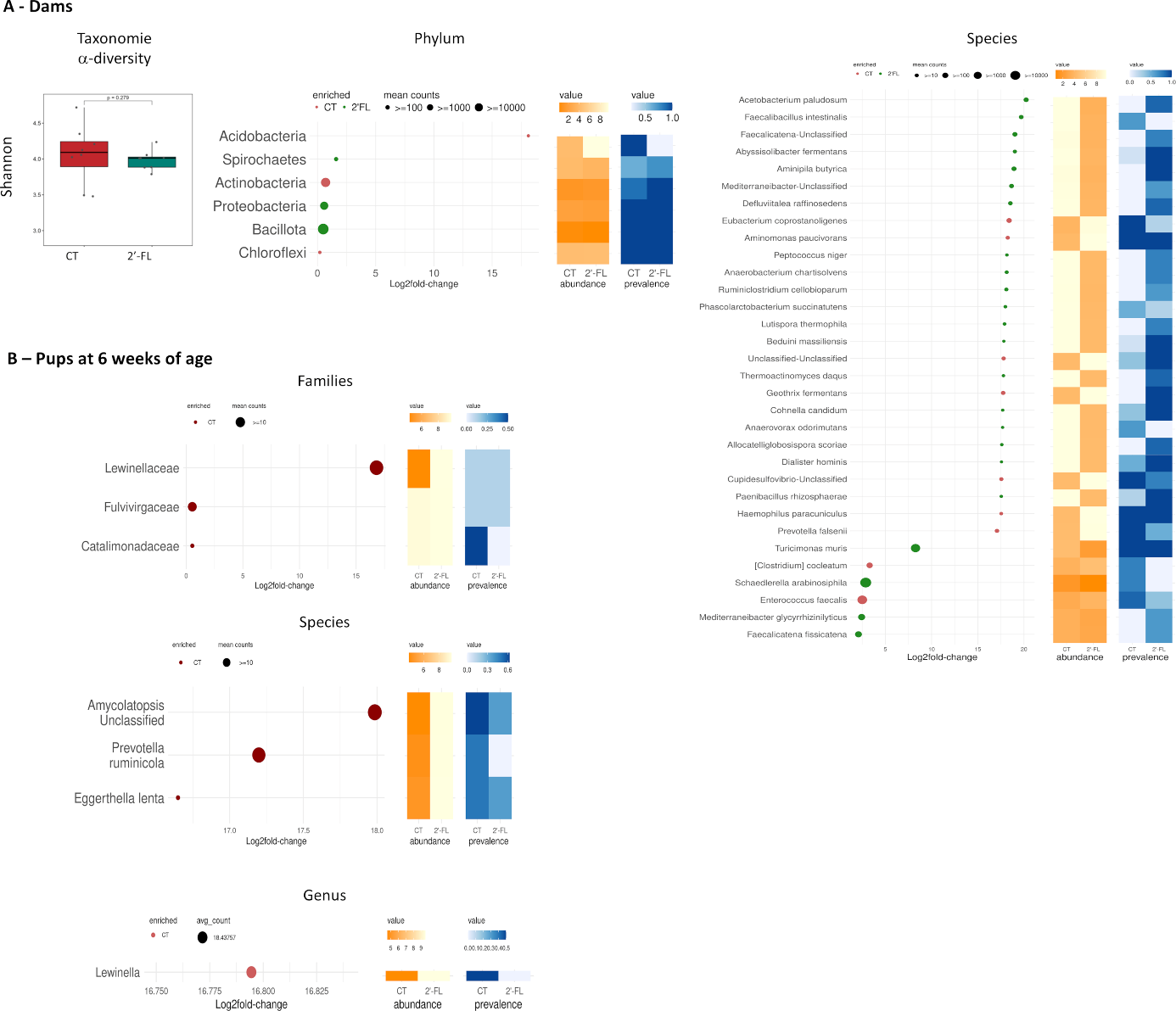


**Figure S3: Shotgun analysis of the gut microbiota of pups at 6 weeks of age. (A)** α- and β-diversity at the enzymatic genes level and α-diversity at the pathway gene level of stools from non- and allergic offspring from mothers fed a control diet (red and yellow respectively) or a 2’-FL enriched diet (green and blue respectively) at 6 weeks of age **(B)** Enzymatic genes relative abundance at the EC3 and EC4 level of stools from non- and allergic offspring from mothers fed a control diet (CT and CT FA respectively) or a 2’-FL enriched diet (2‘-FL and 2’-FL respectively) at 6 weeks of age.


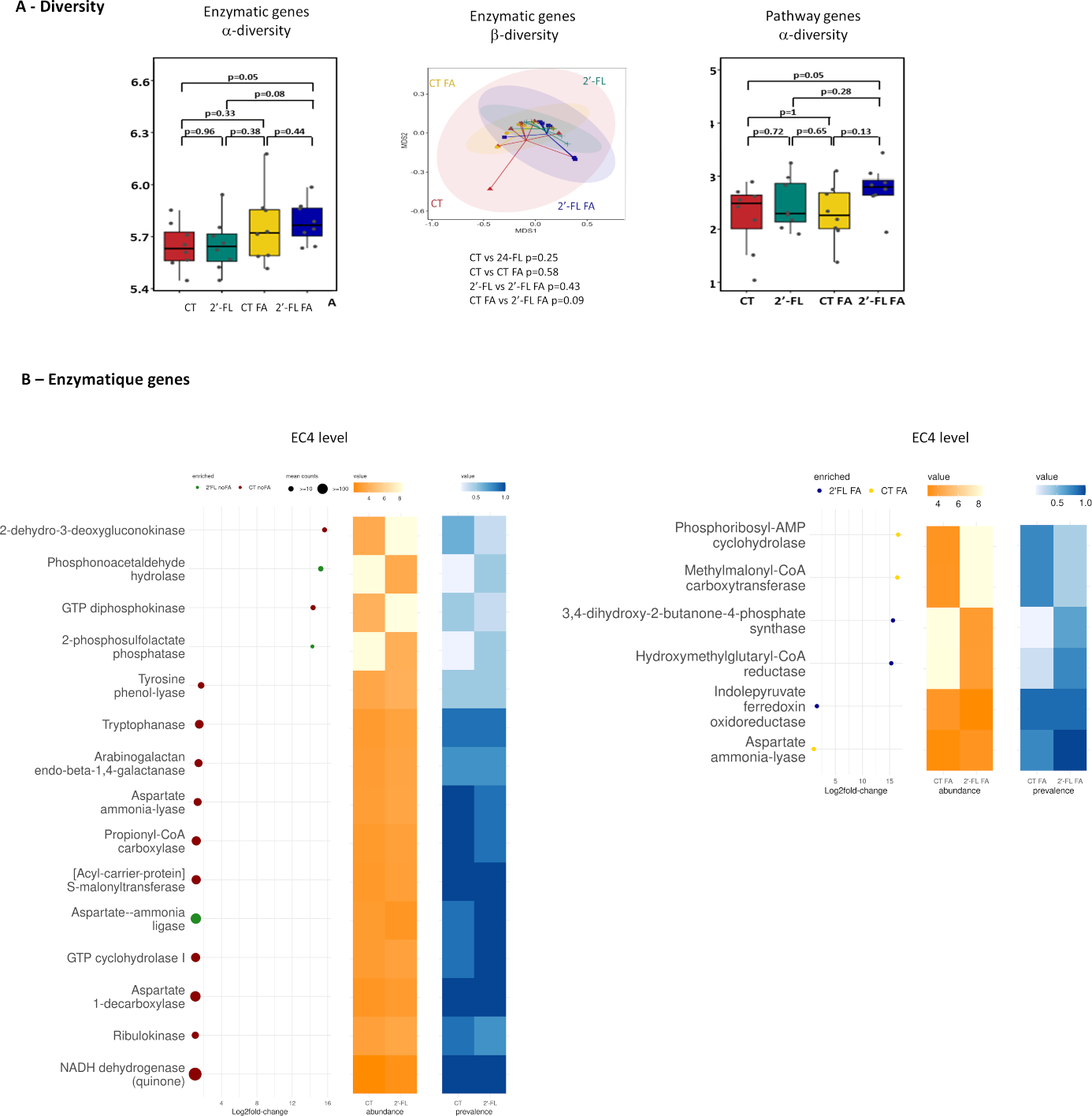


**Figure S4:** **Effects of 2’-FL intake during gestation on the metabolites composition in fetal intestine and amniotic fluid**. Partial least squares discriminant analysis (PLS-DA) of the samples from **(A)** fetal intestine and **(B)** amniotic fluid.


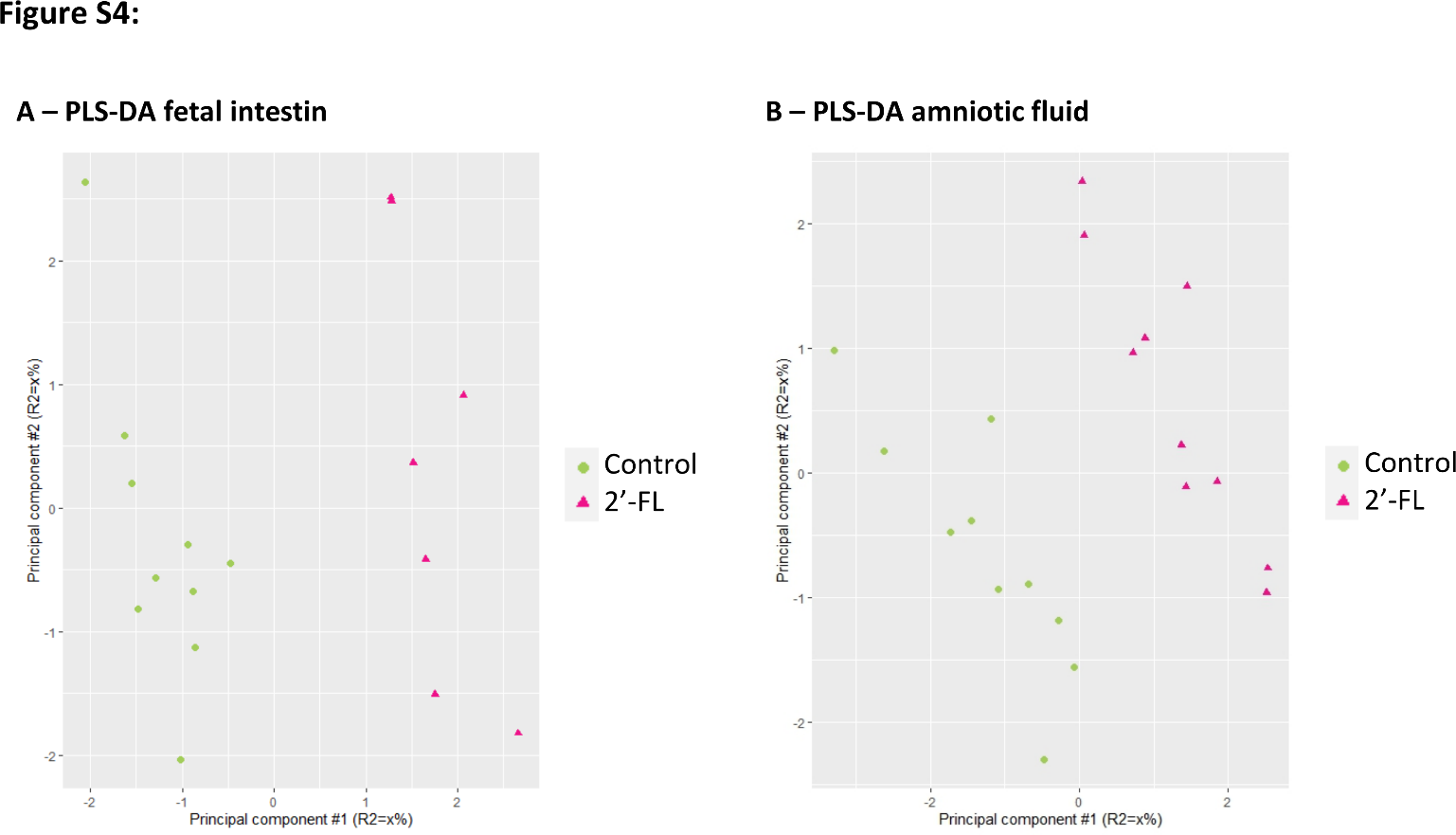


**Figure S5: Gating strategy used for the identification of immune cells in dams, fetus and feto-maternal tissues**. Gating strategy used to identify (**A**) immune B cell sub-populations and (**B**) immune T cell sub-populations.
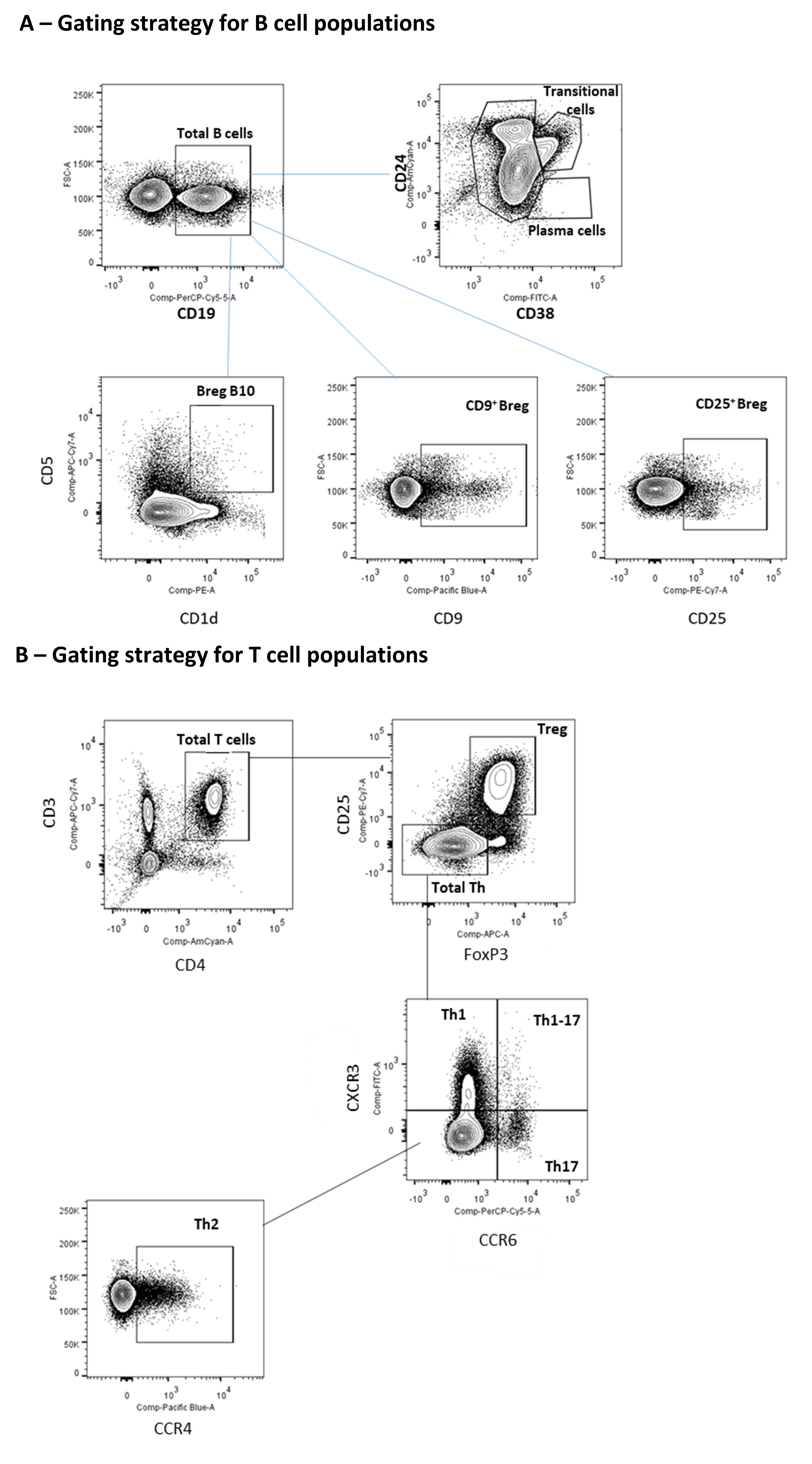


**Figure S6: Contribution on component 2 of variables in the PLS-DA performed immune biomarkers (complement to component 1 presented in Figure 7)**. Panels refer to f immune biomarkers in (**A**) dams bone marrow under control diet (red) or supplemented in 2’-FL (green), (**B**) fetus bone marrow from mother under control diet (red) or supplemented in 2’-FL (green), (**C**) spleen of pups at 3 weeks of age from mother under control diet (red) or supplemented in 2’-FL (green), (**D**) spleen of non-allergic pups at 6 weeks of age from mother under control diet (red) or supplemented in 2’-FL (green), (**E**) spleen of allergic pups at 6 weeks of age from mother under control diet (yellow) or supplemented in 2’-FL (blue). Contributions to components are colored according to the group in which the expression of the variable is maximal based on the median.


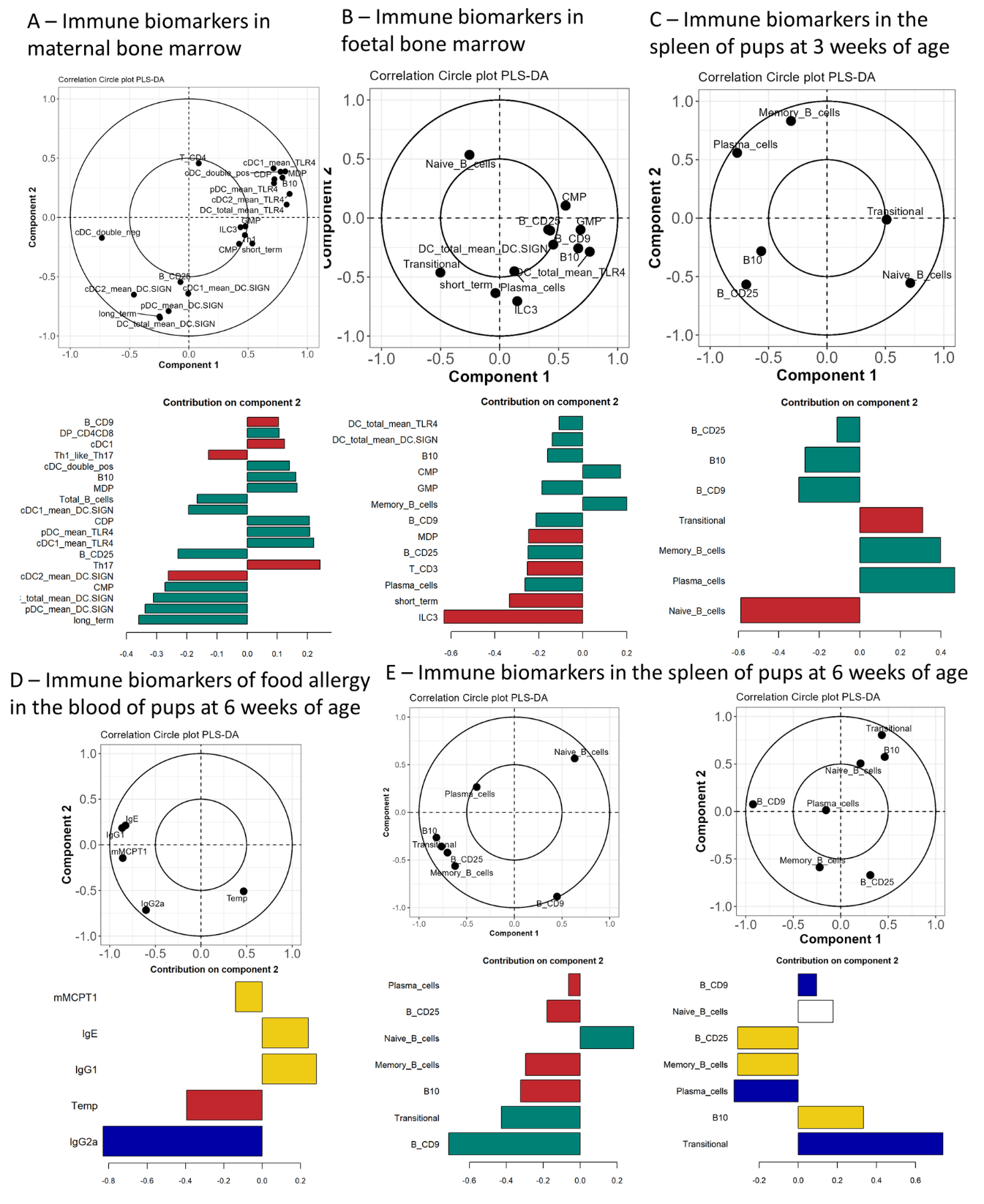


**Table S1: Panel of antibodies for flow cytometry analysis.** Fluorescently labeled anti-mouse antibodies for immune B, T, DC and CMP cells identified by flow cytometry in spleen, mesenteric lymph nodes, bone marrow, intestine and gestational tissues


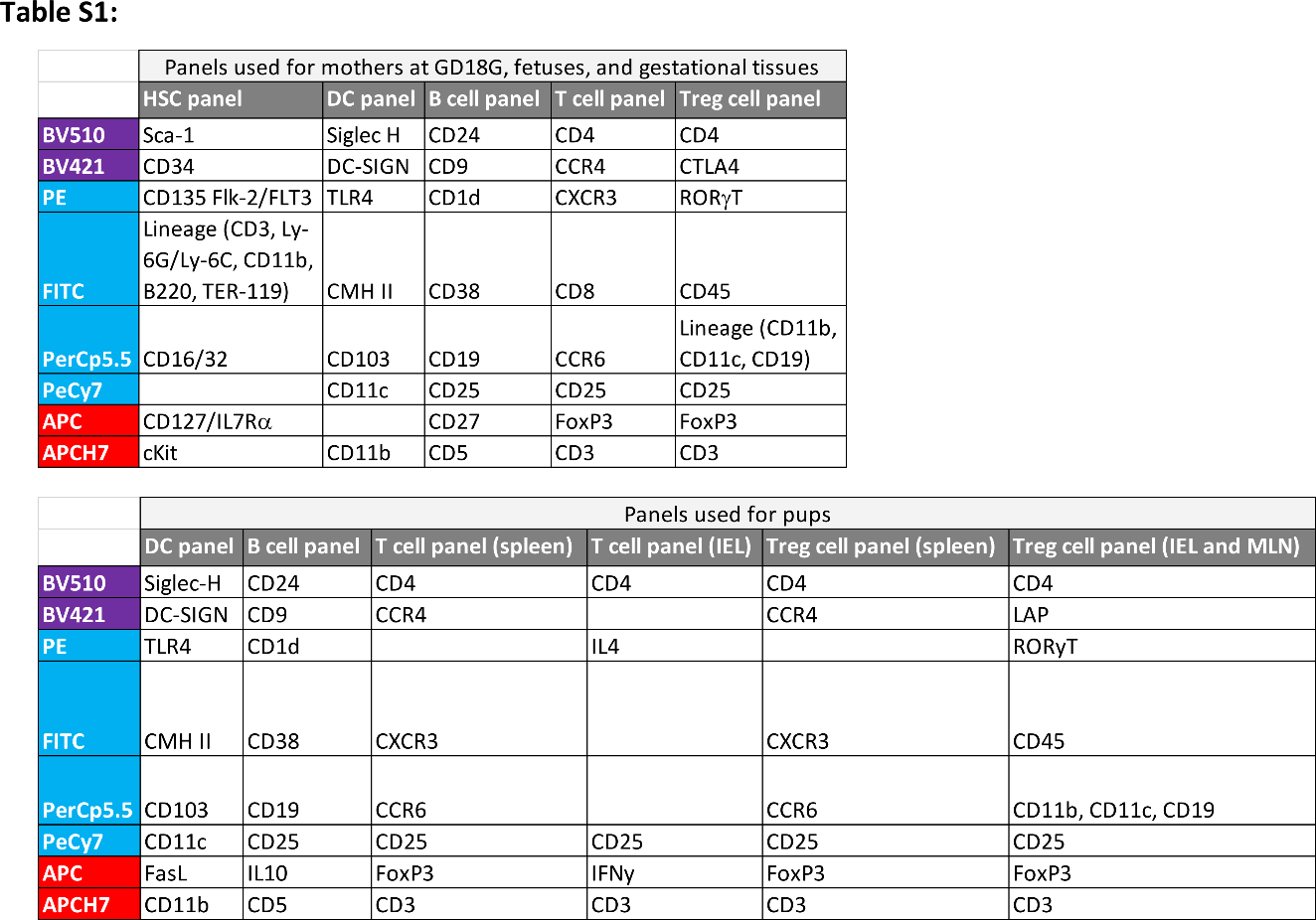

Supplement: Supplemental Material [file KGMI_A_2523813_SM7692.docx]
